# Supplementary figures and images for: Rtf1 HMD domain facilitates global histone H2B monoubiquitination and regulates morphogenesis and virulence in the meningitis-causing pathogen Cryptococcus neoformans
Source: eLife. 2025 May 12;13:RP99229. doi: 10.7554/eLife.99229 (PMC12068867; doi:10.7554/eLife.99229)

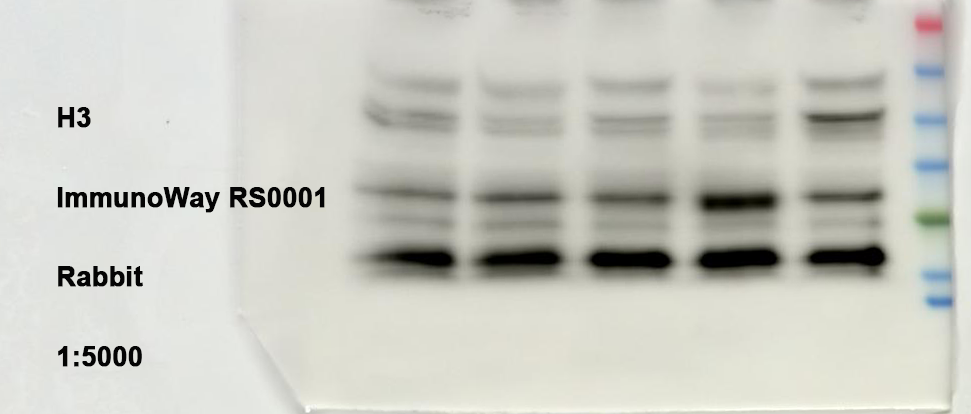

Supplement: Figure 1—source data 1. [file elife-99229-fig1-data1.zip › Figure 1-source data 1/FIG1I-H3.tif]

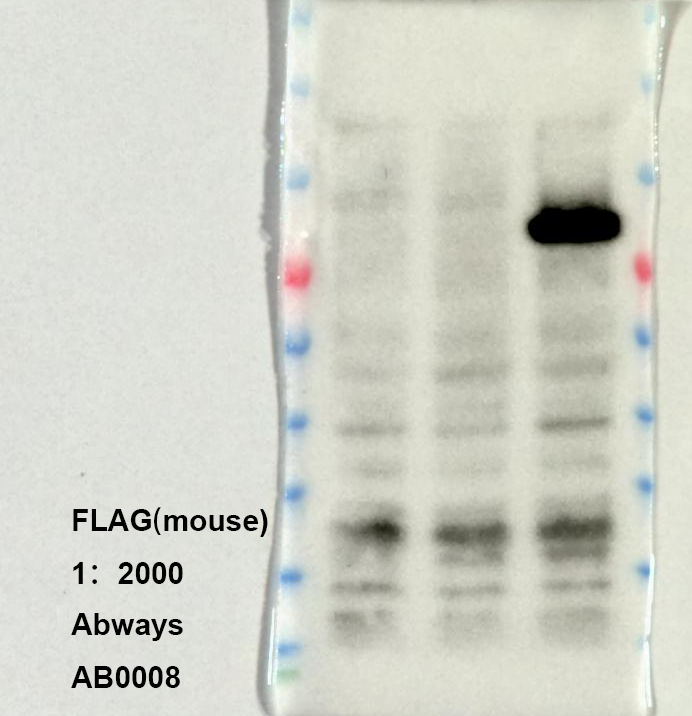

Supplement: Figure 1—source data 1. [file elife-99229-fig1-data1.zip › Figure 1-source data 1/FIG1A-FLAG.tif]

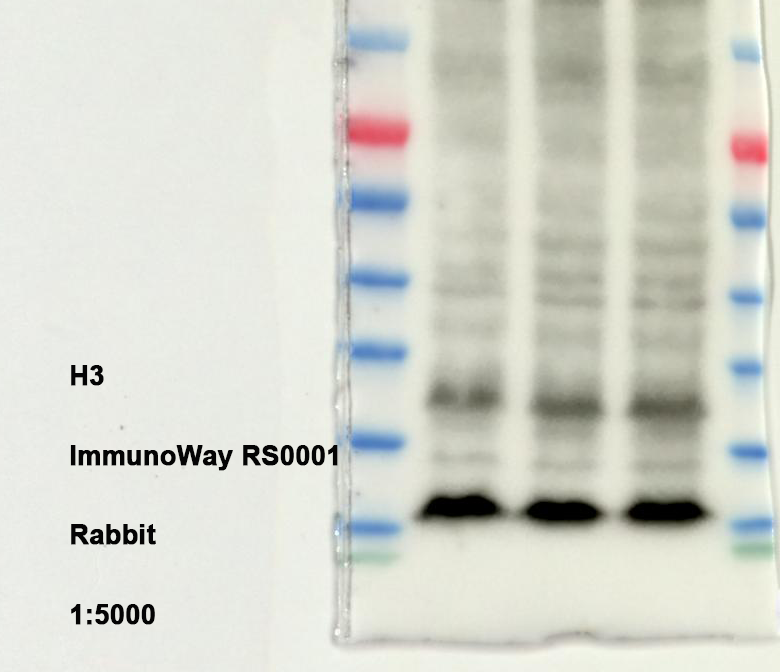

Supplement: Figure 1—source data 1. [file elife-99229-fig1-data1.zip › Figure 1-source data 1/FIG1A-H3.tif]

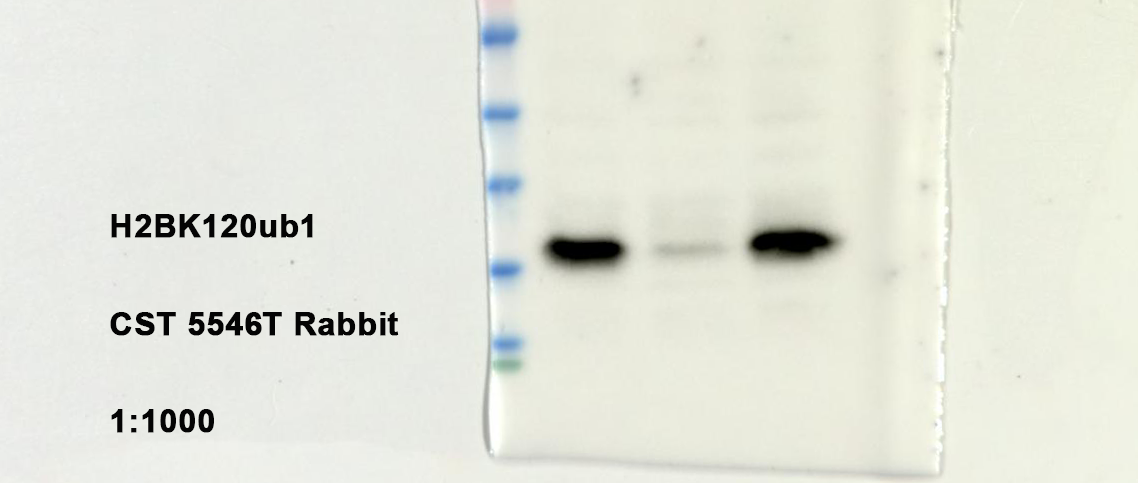

Supplement: Figure 1—source data 1. [file elife-99229-fig1-data1.zip › Figure 1-source data 1/FIG1A-ub1.tif]

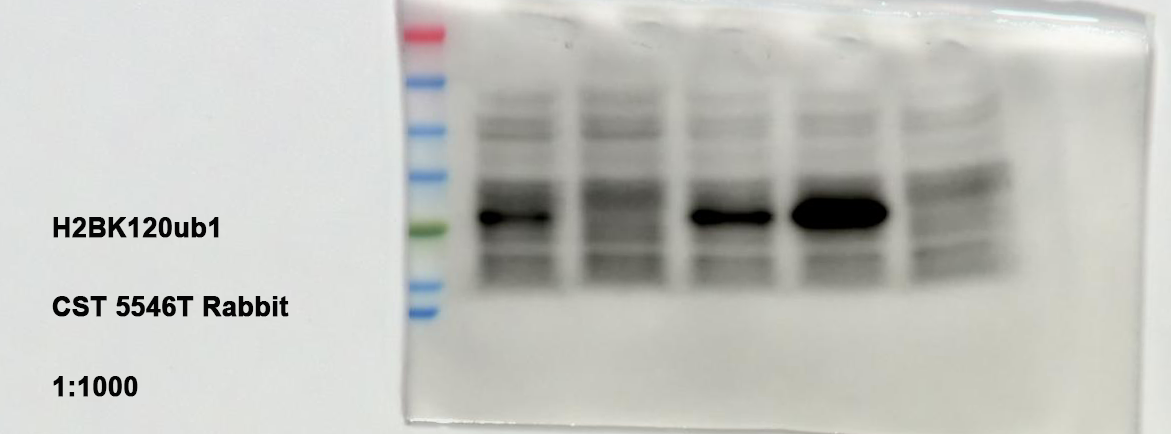

Supplement: Figure 1—source data 1. [file elife-99229-fig1-data1.zip › Figure 1-source data 1/FIG1I-ub1.tif]

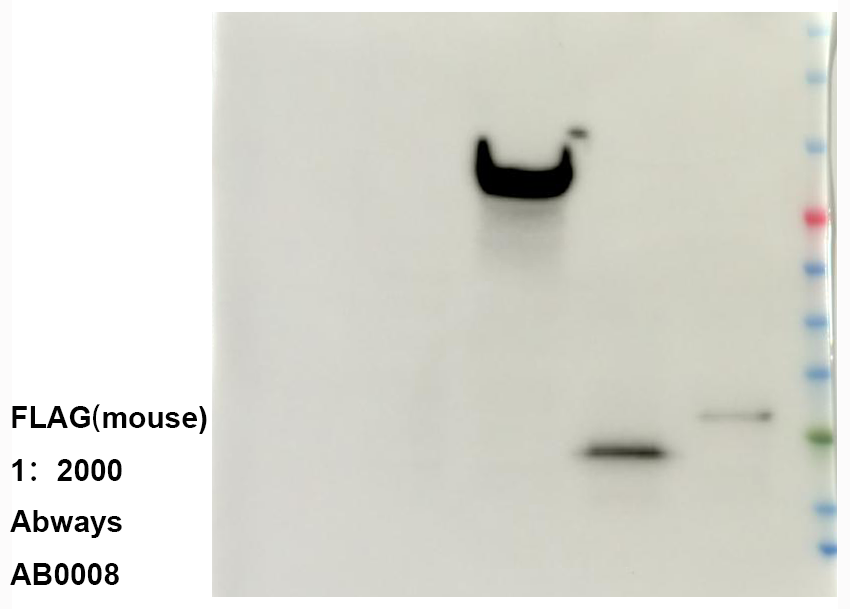

Supplement: Figure 1—source data 1. [file elife-99229-fig1-data1.zip › Figure 1-source data 1/FIG1I-FLAG.tif]

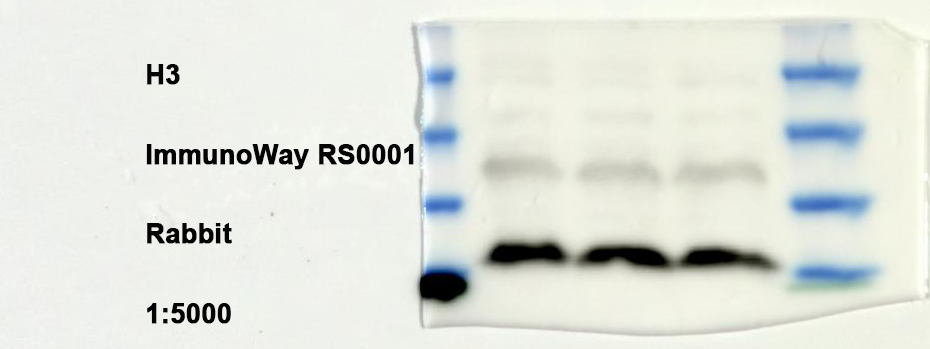

Supplement: Figure 1—source data 1. [file elife-99229-fig1-data1.zip › Figure 1-source data 1/FIG1B-H3.tif]

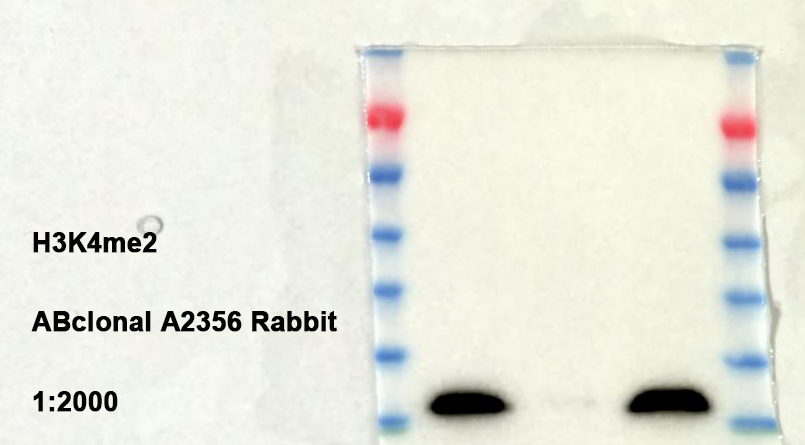

Supplement: Figure 1—source data 1. [file elife-99229-fig1-data1.zip › Figure 1-source data 1/FIG1B-me2.tif]

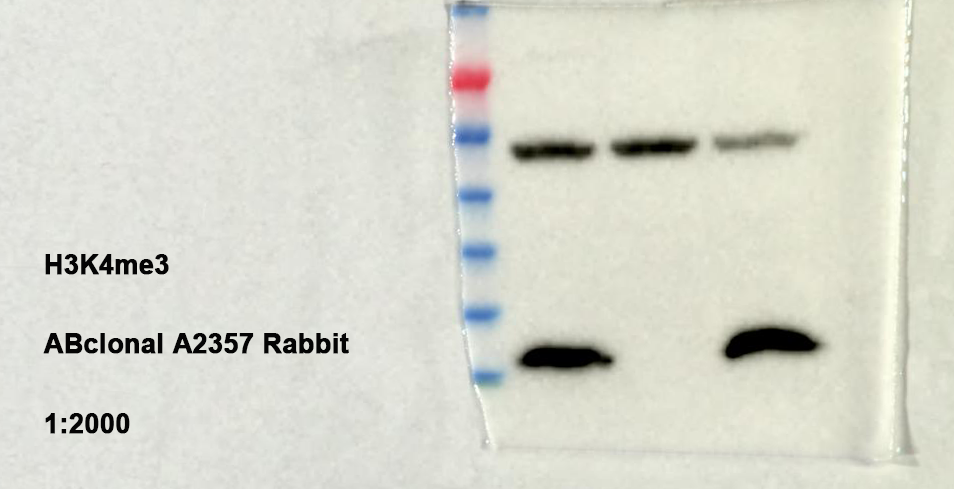

Supplement: Figure 1—source data 1. [file elife-99229-fig1-data1.zip › Figure 1-source data 1/FIG1B-me3.tif]

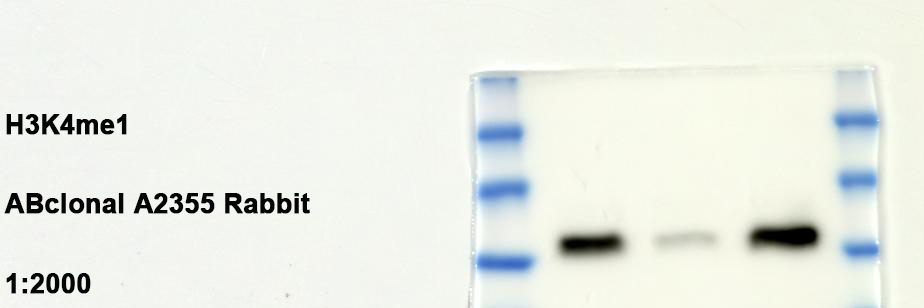

Supplement: Figure 1—source data 1. [file elife-99229-fig1-data1.zip › Figure 1-source data 1/FIG1B-me1.tif]

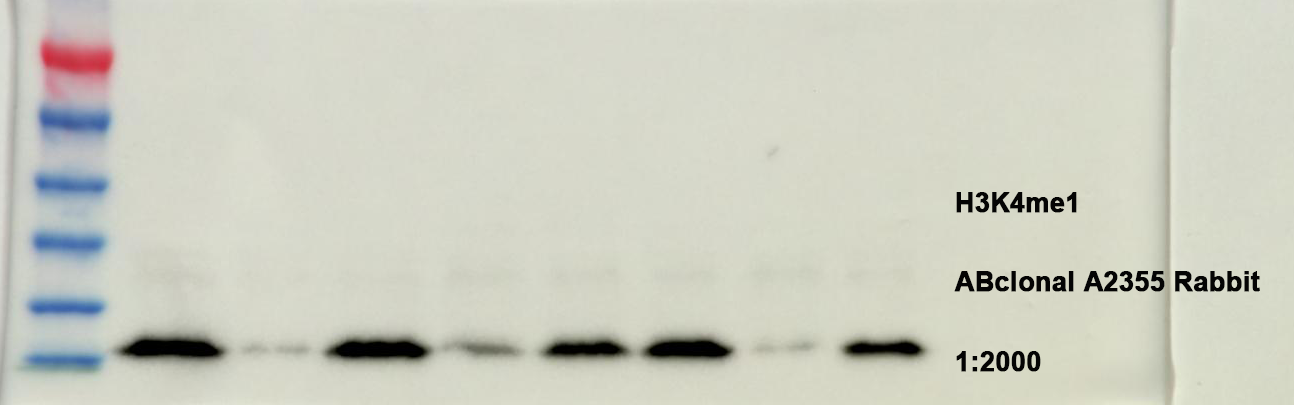

Supplement: Figure 3—source data 1. [file elife-99229-fig3-data1.zip › Figure 3-source data 1/FIG3H-me1.tif]

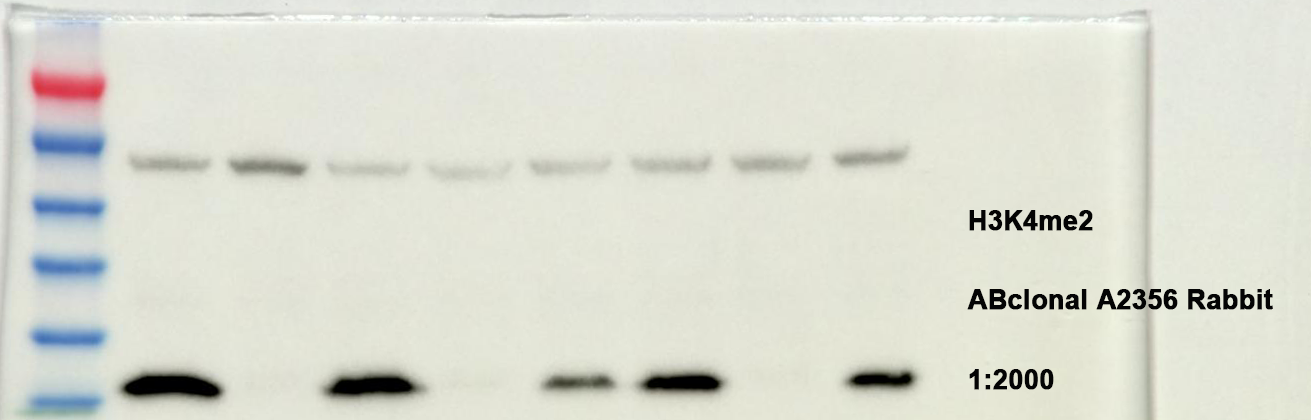

Supplement: Figure 3—source data 1. [file elife-99229-fig3-data1.zip › Figure 3-source data 1/FIG3H-me2.tif]

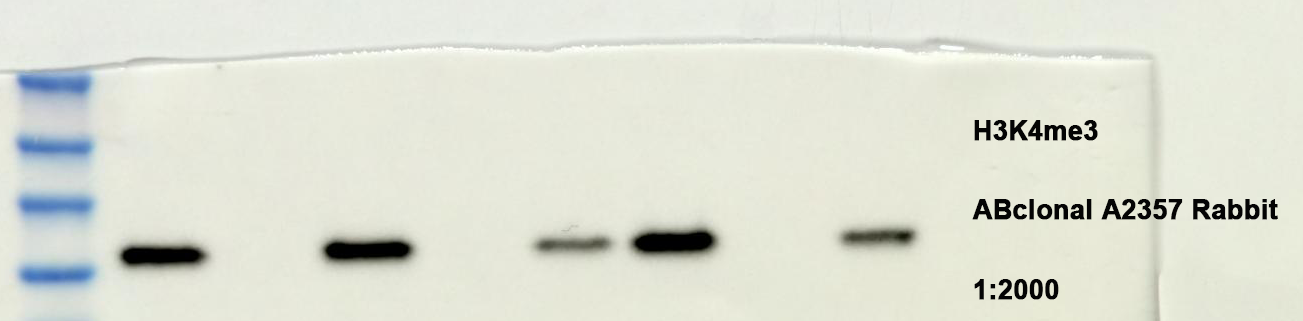

Supplement: Figure 3—source data 1. [file elife-99229-fig3-data1.zip › Figure 3-source data 1/FIG3H-me3.tif]

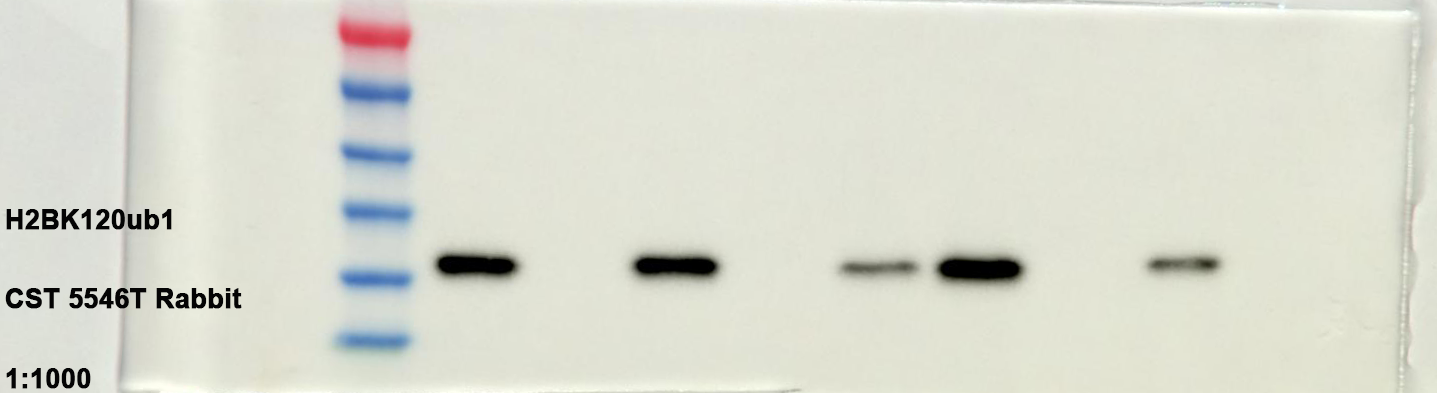

Supplement: Figure 3—source data 1. [file elife-99229-fig3-data1.zip › Figure 3-source data 1/FIG3H-ub1.tif]

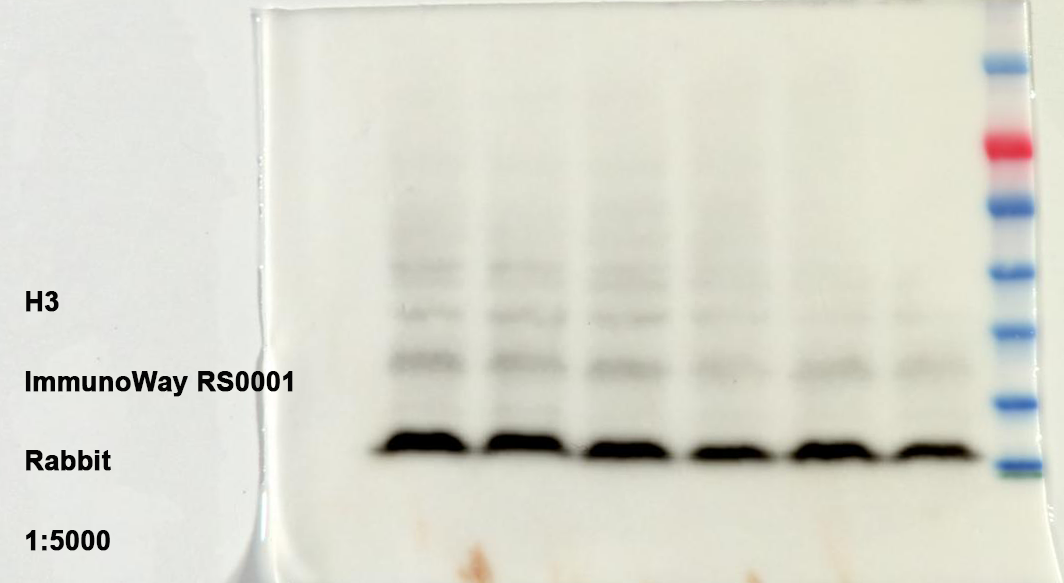

Supplement: Figure 3—source data 1. [file elife-99229-fig3-data1.zip › Figure 3-source data 1/FIG3C-H3.tif]

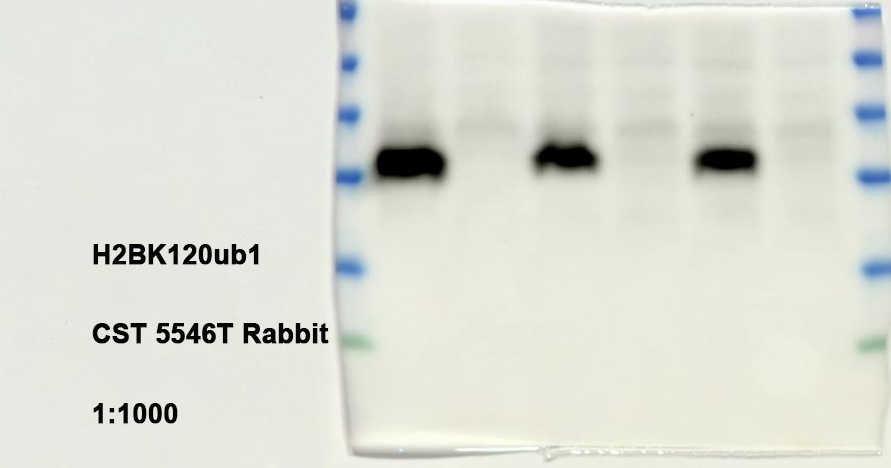

Supplement: Figure 3—source data 1. [file elife-99229-fig3-data1.zip › Figure 3-source data 1/FIG3C-ub1.tif]

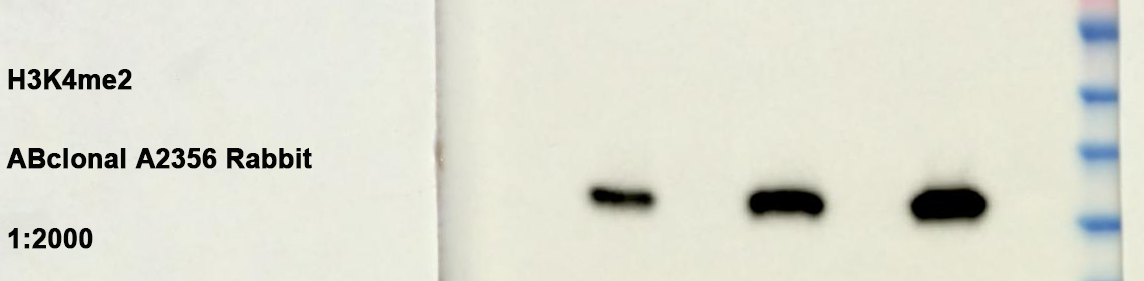

Supplement: Figure 3—source data 1. [file elife-99229-fig3-data1.zip › Figure 3-source data 1/FIG3C-me2.tif]

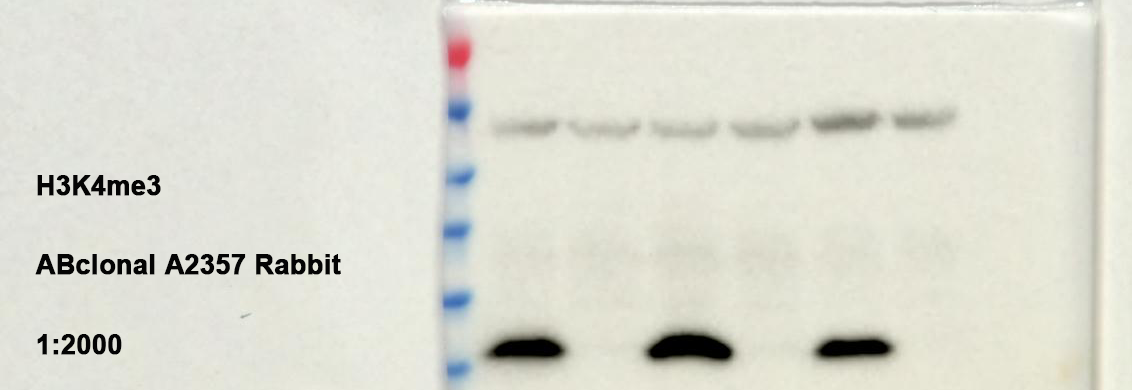

Supplement: Figure 3—source data 1. [file elife-99229-fig3-data1.zip › Figure 3-source data 1/FIG3C-me3.tif]

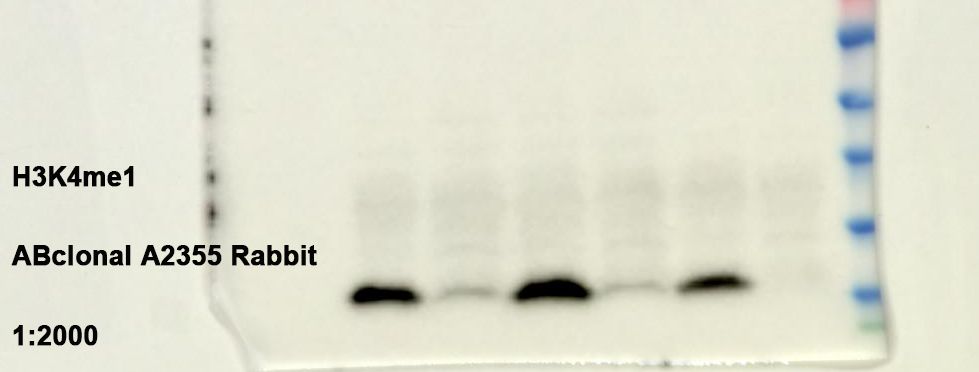

Supplement: Figure 3—source data 1. [file elife-99229-fig3-data1.zip › Figure 3-source data 1/FIG3C-me1.tif]

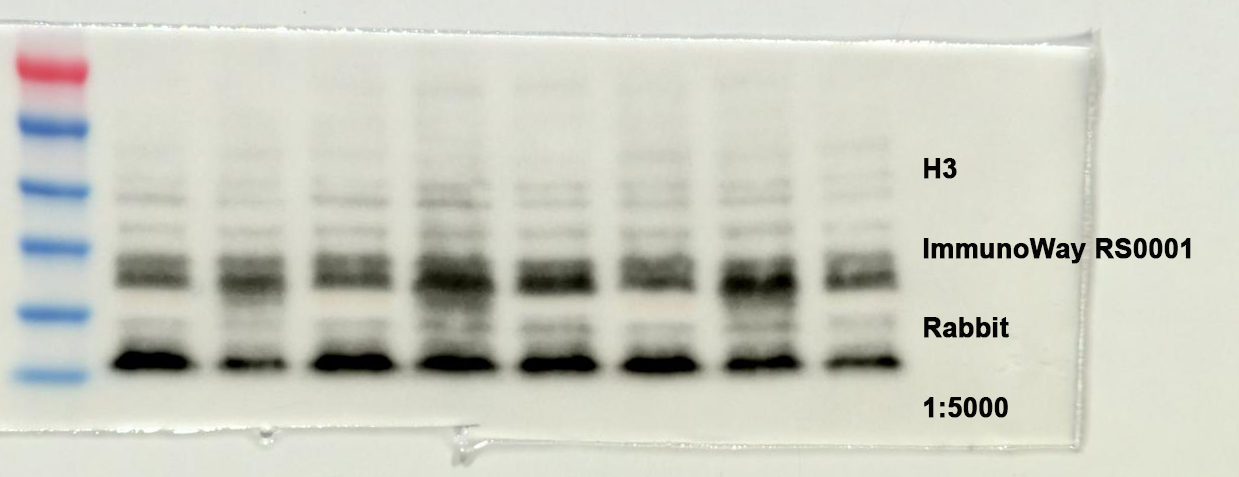

Supplement: Figure 3—source data 1. [file elife-99229-fig3-data1.zip › Figure 3-source data 1/FIG3H-H3.tif]

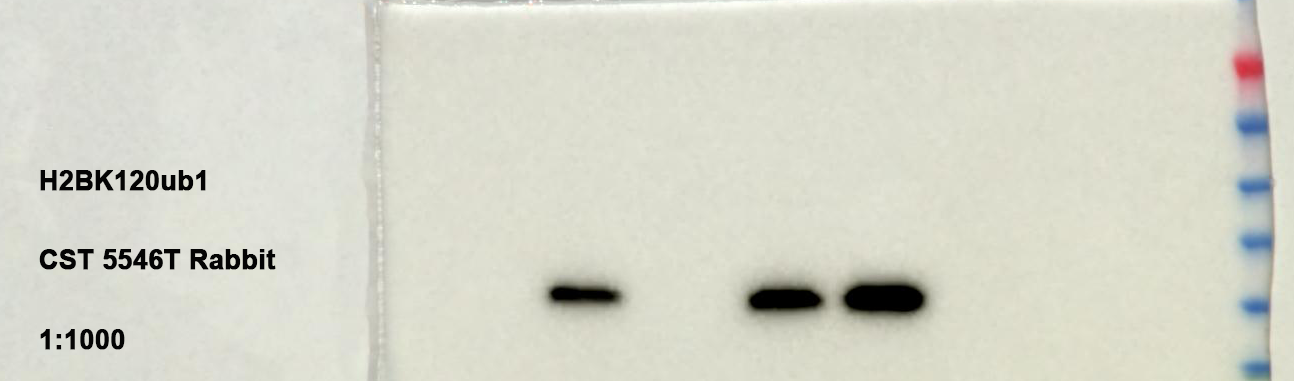

Supplement: Figure 4—source data 1. [file elife-99229-fig4-data1.zip › Figure 4-source data 1/FIG4A-ub1.tif]

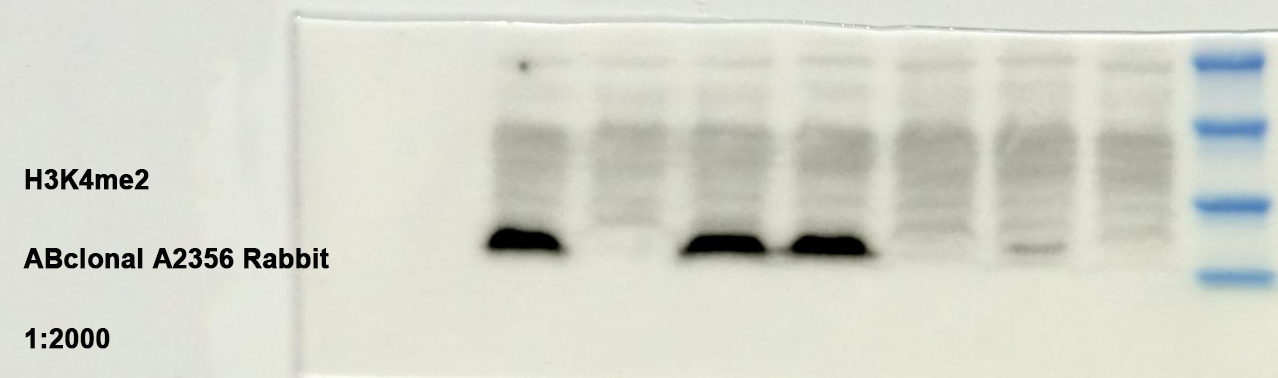

Supplement: Figure 4—source data 1. [file elife-99229-fig4-data1.zip › Figure 4-source data 1/FIG4A-me2.tif]

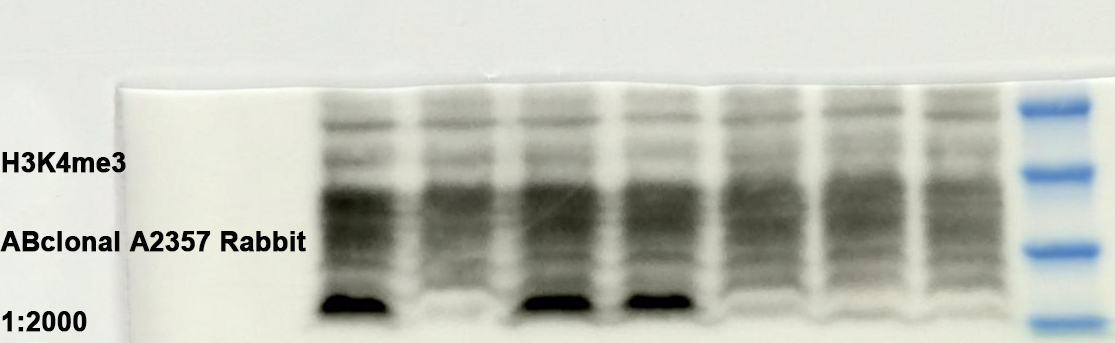

Supplement: Figure 4—source data 1. [file elife-99229-fig4-data1.zip › Figure 4-source data 1/FIG4A-me3.tif]

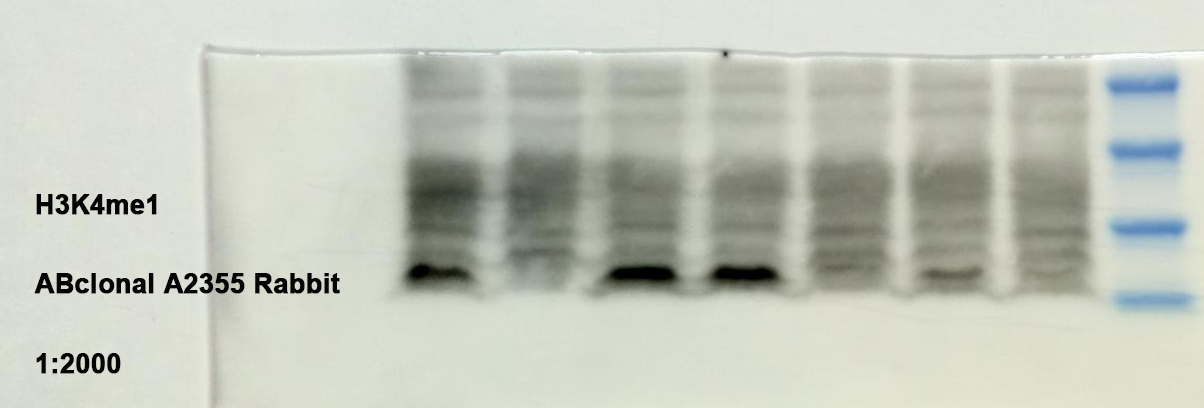

Supplement: Figure 4—source data 1. [file elife-99229-fig4-data1.zip › Figure 4-source data 1/FIG4A-me1.tif]

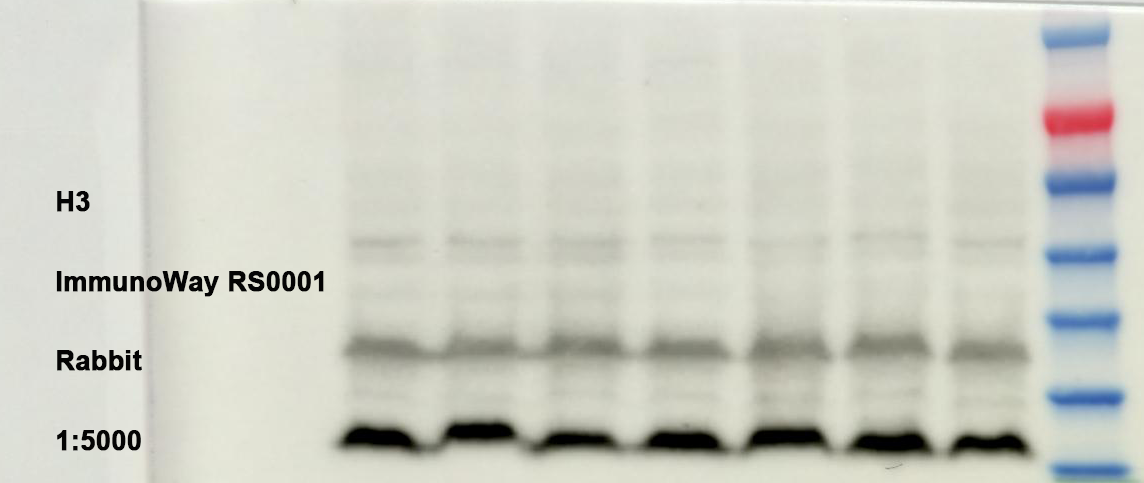

Supplement: Figure 4—source data 1. [file elife-99229-fig4-data1.zip › Figure 4-source data 1/FIG4A-H3.tif]
