# Supplementary material for: Rtf1 HMD domain facilitates global histone H2B monoubiquitination and regulates morphogenesis and virulence in the meningitis-causing pathogen Cryptococcus neoformans
Source: eLife. 2025 May 12;13:RP99229. doi: 10.7554/eLife.99229 (PMC12068867; doi:10.7554/eLife.99229)
Supplement: Figure 1—source data 2. [file elife-99229-fig1-data2.zip › Figure 1-source data 2/Figure source data 2.pptx]

## Slide 1
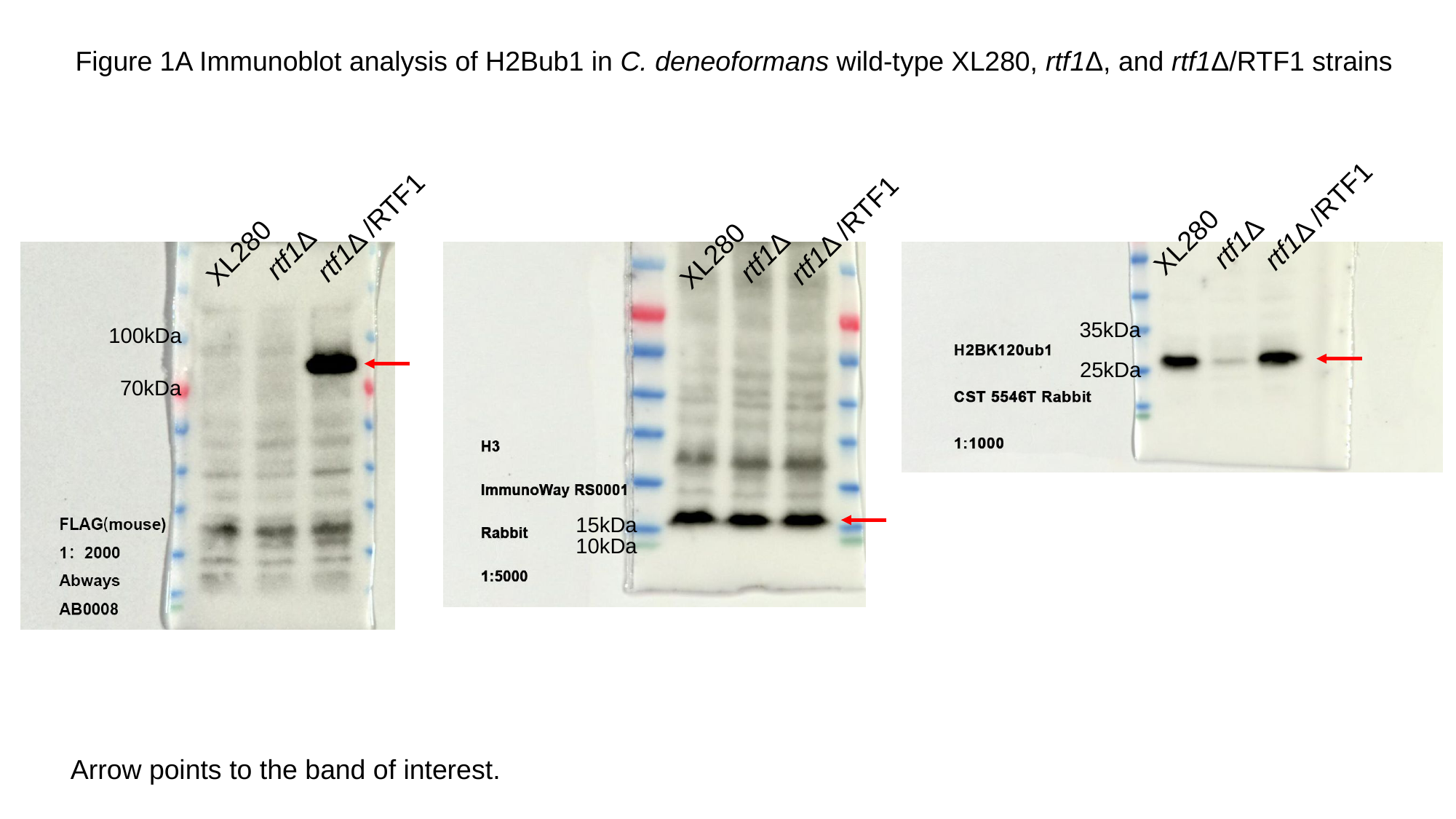

Figure 1A Immunoblot analysis of H2Bub1 in C. deneoformans wild-type XL280, rtf1Δ, and rtf1Δ/RTF1 strains
rtf1Δ /RTF1
rtf1Δ /RTF1
rtf1Δ /RTF1
XL280
rtf1Δ
XL280
rtf1Δ
XL280
rtf1Δ
35kDa
100kDa
25kDa
70kDa
15kDa
10kDa
Arrow points to the band of interest.

## Slide 2
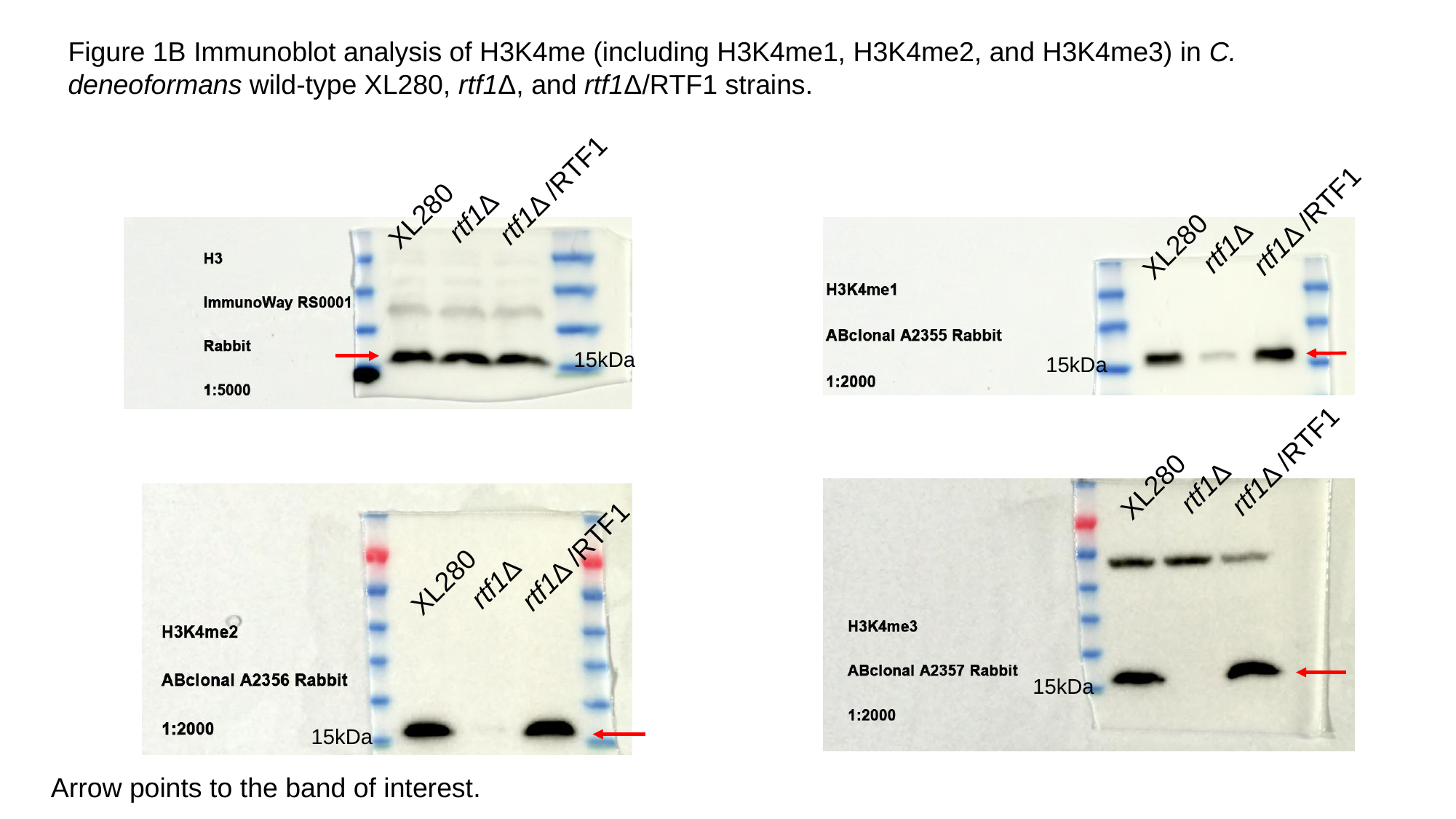

Figure 1B Immunoblot analysis of H3K4me (including H3K4me1, H3K4me2, and H3K4me3) in C. deneoformans wild-type XL280, rtf1Δ, and rtf1Δ/RTF1 strains.
rtf1Δ /RTF1
XL280
rtf1Δ
rtf1Δ /RTF1
XL280
rtf1Δ
15kDa
15kDa
rtf1Δ /RTF1
XL280
rtf1Δ
rtf1Δ /RTF1
XL280
rtf1Δ
15kDa
15kDa
Arrow points to the band of interest.

## Slide 3
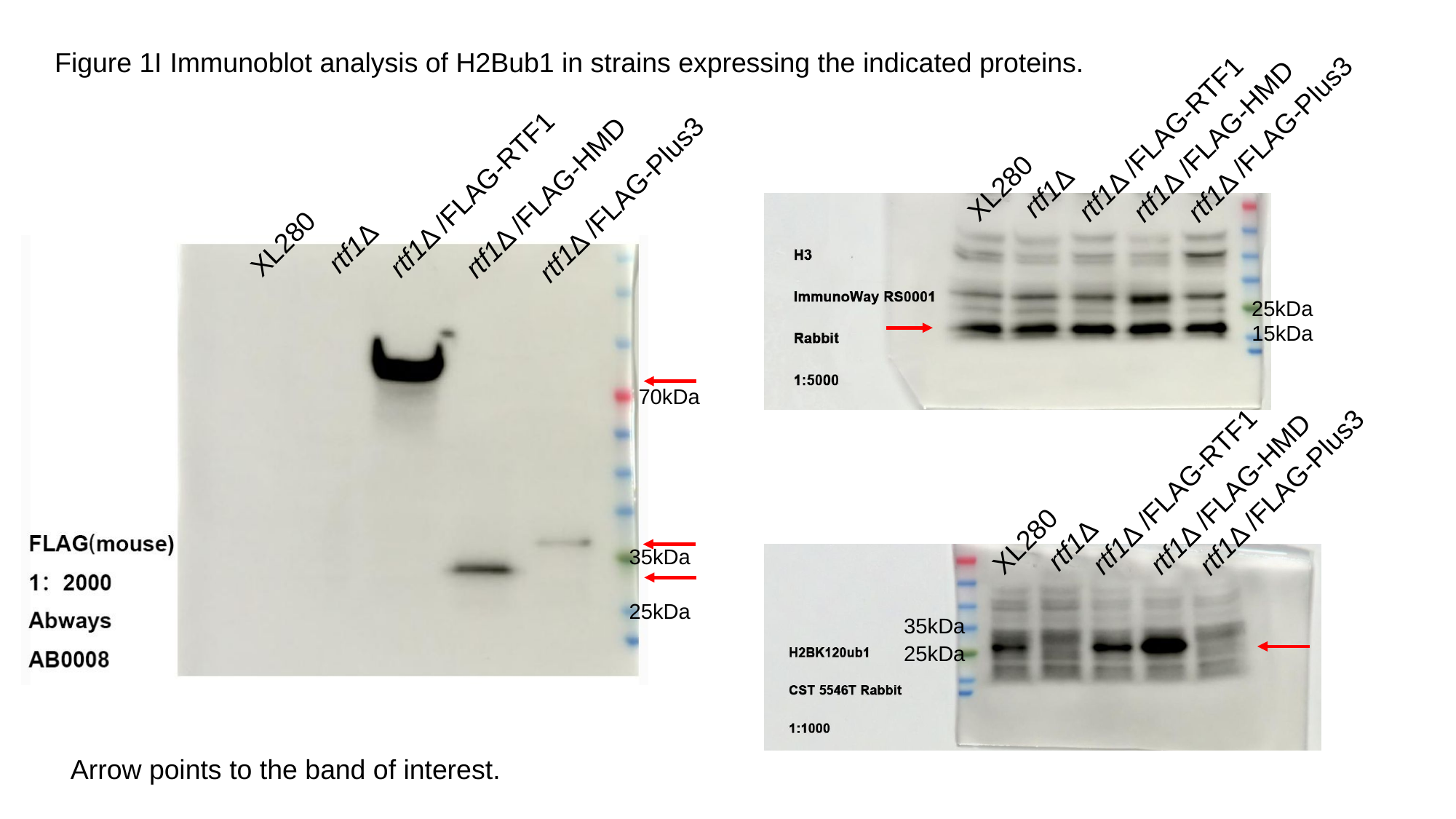

Figure 1I Immunoblot analysis of H2Bub1 in strains expressing the indicated proteins.
rtf1Δ /FLAG-RTF1
rtf1Δ /FLAG-Plus3
rtf1Δ /FLAG-HMD
XL280
rtf1Δ
rtf1Δ /FLAG-RTF1
rtf1Δ /FLAG-HMD
rtf1Δ /FLAG-Plus3
XL280
rtf1Δ
25kDa
15kDa
70kDa
rtf1Δ /FLAG-RTF1
rtf1Δ /FLAG-Plus3
rtf1Δ /FLAG-HMD
XL280
rtf1Δ
35kDa
25kDa
35kDa
25kDa
Arrow points to the band of interest.
