# Supplementary material for: Rtf1 HMD domain facilitates global histone H2B monoubiquitination and regulates morphogenesis and virulence in the meningitis-causing pathogen Cryptococcus neoformans
Source: eLife. 2025 May 12;13:RP99229. doi: 10.7554/eLife.99229 (PMC12068867; doi:10.7554/eLife.99229)
Supplement: Figure 4—source data 2. [file elife-99229-fig4-data2.zip › Figure 4-source data 2/Figure 4-source data 2.pptx]

## Slide 1
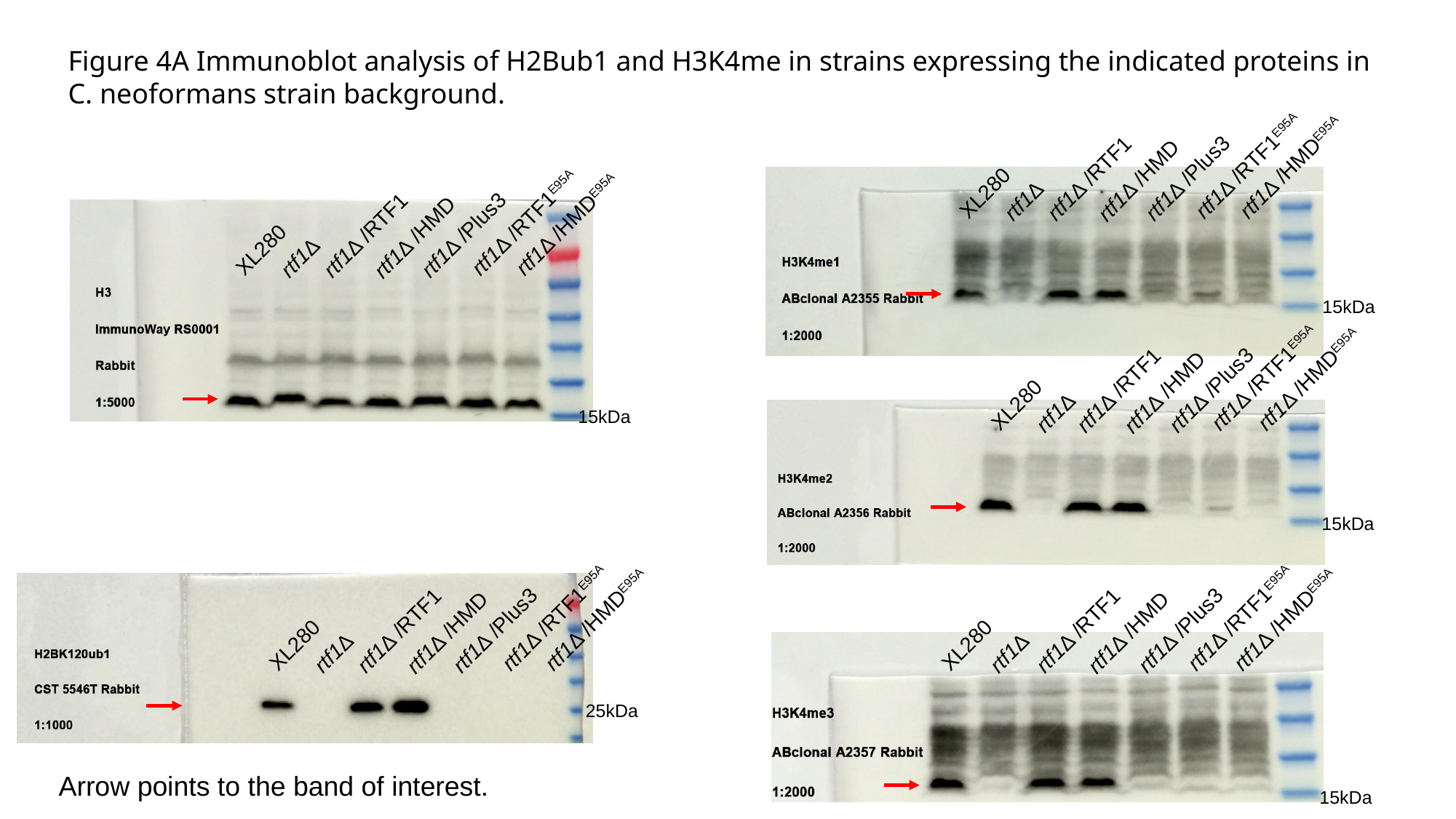

Figure 4A Immunoblot analysis of H2Bub1 and H3K4me in strains expressing the indicated proteins in C. neoformans strain background.
rtf1Δ /RTF1E95A
rtf1Δ /HMDE95A
rtf1Δ /Plus3
rtf1Δ /RTF1
rtf1Δ /HMD
XL280
rtf1Δ
rtf1Δ /RTF1E95A
rtf1Δ /HMDE95A
rtf1Δ /Plus3
rtf1Δ /RTF1
rtf1Δ /HMD
XL280
rtf1Δ
15kDa
rtf1Δ /RTF1E95A
rtf1Δ /HMDE95A
rtf1Δ /Plus3
rtf1Δ /RTF1
rtf1Δ /HMD
XL280
rtf1Δ
15kDa
15kDa
rtf1Δ /RTF1E95A
rtf1Δ /RTF1E95A
rtf1Δ /HMDE95A
rtf1Δ /HMDE95A
rtf1Δ /Plus3
rtf1Δ /Plus3
rtf1Δ /RTF1
rtf1Δ /RTF1
rtf1Δ /HMD
rtf1Δ /HMD
XL280
XL280
rtf1Δ
rtf1Δ
25kDa
Arrow points to the band of interest.
15kDa
