# Supplementary material for: Rtf1 HMD domain facilitates global histone H2B monoubiquitination and regulates morphogenesis and virulence in the meningitis-causing pathogen Cryptococcus neoformans
Source: eLife. 2025 May 12;13:RP99229. doi: 10.7554/eLife.99229 (PMC12068867; doi:10.7554/eLife.99229)
Supplement: Supplementary file 1. [file elife-99229-supp1.docx]

| **Strain name** | **Genotype** | **Background** | **Sources and comments** |
| --- | --- | --- | --- |
| XL280α | WT | XL280α | [51] |
| XC27 | *rtf1::NAT* | XL280α | This study |
| XC111 | *rtf1::NAT, P_CTR4_-3xFLAG-RTF1-NEO* | XL280α | This study |
| YJ3 | *rtf1::NAT, P_TEF1_-HMD-2xFLAG-HYG* | XL280α | This study |
| TW3 | *rtf1::NAT, P_TEF1_-Plus3-2x FLAG-HYG* | XL280α | This study |
| YJ53 | *rtf1::NAT, P_CTR4_-3xFLAG-RTF1-E95A-NEO* | XL280α | This study |
| YL27 | *rtf1::NAT, P_TEF1_-HMD-E95A-2xFLAG-HYG* | XL280α | This study |
| YJ55 | *rtf1::NAT, P_CTR4_-3xFLAG-RTF1-F118A-NEO* | XL280α | This study |
| YL30 | *rtf1::NAT, P_TEF1_-HMD-F118A-2xFLAG-HYG* | XL280α | This study |
| YJ70 | *rtf1::NAT,P_H3_-RGS2membrane-mNeonGreen-RTF1-NEO* | XL280α | This study |
| YJ73 | *rtf1::NAT,P_H3_-RGS2membrane-mNeonGreen-HMD-NEO* | XL280α | This study |
| YJ74 | *rtf1::NAT,P_TEF1_-RTF1-mNeonGreen-NEO* | XL280α | This study |
| YJ15 | *rtf1::NAT, P_TEF1_-NLS-RTF1-HMD-mNeonGreen-NEO* | XL280α | This study |
| XL280**a** | WT | XL280**a** | [51] |
| XC129 | *rtf1::NAT* | XL280**a** | This study |
| H99α | WT | H99α | Lab stock |
| RL226 | *rtf1::NAT* | H99α | Madhani’s deletion set |
| YJ62 | *rtf1::NAT, P_CTR4_-3xFLAG-RTF1-NEO* | H99α | This study |
| YJ64 | *rtf1::NAT, P_TEF1_-HMD-2xFLAG-HYG* | H99α | This study |
| YJ67 | *rtf1::NAT, P_TEF1_-Plus3-2xFLAG-HYG* | H99α | This study |
| YJ78 | *rtf1::NAT, P_CTR4_-3xFLAG-RTF1-E95A-NEO* | H99α | This study |
| YJ82 | *rtf1::NAT, P_TEF1_-HMD-E95A-2xFLAG-HYG* | H99α | This study |

**Strains, plasmids, primers and antibodies used in this study.**

| **Plasmid** | **Genotype** | **Sources** |
| --- | --- | --- |
| pYZ175 | P*_CTR4_-3×FLAG-NEO* | This study |
| pYZ41 | P*_TEF1_-2xFLAG-HYG* | This study |
| pYZ25 | P*_TEF1_-mNeonGreen-NEO* | This study |
| pYZ281 | P*_CTR4_-3xFLAG-RTF1-NEO* | This study |
| pYZ255 | P*_TEF1_-HMD-core-2xFLAG-NEO* | This study |
| pYZ278 | P*_TEF1_-ATG-NLS-Plus3-2xFLAG-HYG* | This study |
| pYZ463 | P*_CTR4_-3xFLAG-RTF1-E95A-NEO* | This study |
| pYZ455 | P*_TEF1_-HMD-E95A-2xFLAG-HYG* | This study |
| pYZ467 | P*_CTR4_-3xFLAG-RTF1-F118A-NEO* | This study |
| pYZ459 | P*_TEF1_-HMD-F118A-2xFLAG-HYG* | This study |
| pYZ317 | P*_H3_-RGS2membrane-mNeonGreen-NEO* | This study |
| pYZ506 | P*_H3_-RGS2membrane-mNeonGreen-RTF1-NEO* | This study |
| pYZ509 | P*_H3_-RGS2membrane-mNeonGreen-HMD-NEO* | This study |
| pYZ554 | P*_TEF1_-RTF1-mNeonGreen-NEO* | This study |
| pYZ269 | P*_TEF1_-NLS-HMD-mNeonGreen-NEO* | This study |

| **Primer name** | **Sequence (5’ to 3’)** | **Description** |
| --- | --- | --- |
| M13F | GTAAAACGACGGCCAGT | NAT/NEO/HYG cassette and TRACE constructs from plasmid |
| M13R | CAGGAAACAGCTATGAC | NAT/NEO/HYG cassette and TRACE constructs from plasmid |
| ZhaoLab0003/YZ | TTGGATGCTGGATGCTGGGT | NAT-F |
| ZhaoLab0004/YZ | CCGTCTTCACCTGCATCTGATT | NAT-split-R |
| ZhaoLab0015/YZ | TGTGGATGCTGGCGGAGGATA | NAT-split-R for positive PCR of mutant |
| ZhaoLab0192/YZ | AACTGAGATACCTACAGCGTGAG | gRNA-scaffold-Far-R |
| ZhaoLab0193/YZ | ACTCCCTGGTCCCATCCCT | CnU6-Far-F |
| ZhaoLab0011/YZ | CCATCGATTTGCATTAGAACTAAAAACAAAGCA | U6 promoter NF |
| ZhaoLab0012/YZ | CCGCTCGAGTAAAACAAAAAAGCACCGAC | gRNA scaffold NR |
| ZhaoLab0054/YZ | GATAGATACTGAGGAGGACAT | PGPD1_F for Cas9 |
| ZhaoLab0055/YZ | GGGCCCCTCTTCACGTGG | TGPD1_R for Cas9 |
| ZhaoLab0013/YZ | AGACTCCACAGCCTAAGATCAACAGTATACCCTGCCGGTG | *SH2* gRNA, paired with 0193  (U6 promoter R) |
| ZhaoLab0014/YZ | GATCTTAGGCTGTGGAGTCTGTTTTAGAGCTAGAAATAGCAAGTT | *SH2* gRNA, paired with 0192  (gRNA scaffold F) |
| ZhaoLab0105/YZ | GTTCTCTGACCCAAAACATCGTTTTAGAGCTAGAAATAGCAAGTT | *SH3* gRNA, paired with 0193  (U6 promoter R) |
| ZhaoLab0106/YZ | GATGTTTTGGGTCAGAGAACAACAGTATACCCTGCCGGTG | *SH3* gRNA, paired with 0192  (gRNA scaffold F) |
| ZhaoLab0188/YZ | CGAAGGATGGTTGTCGCTC | screening insertion into SH3 in serotype D |
| ZhaoLab0189/YZ | GTATCGTCTTGCTCTTCATTCC | screening insertion into SH3 in serotype D |
| ZhaoLab0190/YZ | GTTGTTTCAGGCCTGCGGATG | screening insertion into SH2 in serotype A |
| ZhaoLab0191/YZ | GACTCATTCCTATGCCGTTC | screening insertion into SH2 in serotype A |
| ZhaoLab0044/YZ | TCTGACGCTGCGCCTTTG | *RTF1* deletion in XL280 LF |
| ZhaoLab0045/YZ | ctggccgtcgttttacATGGGATGCTGATGAGATTGCT | *RTF1* deletion in XL280 LR |
| ZhaoLab0046/YZ | GCTTCGGGCACCACTAAC | *RTF1* deletion in XL280 NLF |
| ZhaoLab0047/YZ | GtcatagctgtttcctgGGGAGAAGGATGACTACGA | *RTF1* deletion in XL280 RF |
| ZhaoLab0048/YZ | AAAGCGAACTGTGGACGA | *RTF1* deletion in XL280 RR |
| ZhaoLab0049/YZ | GACAAGAAAAGAAACCGA | *RTF1* deletion in XL280 NRR |
| ZhaoLab0050/YZ | agcatggtaggccaaagtacGTTTTAGAGCTAGAAATAGCAAGTT | *RTF1* deletion in XL280 gRNA, paired with 0192 (gRNA scaffold F) |
| ZhaoLab0051/YZ | GTACTTTGGCCTACCATGCTAACAGTATACCCTGCCGGTG | *RTF1* deletion in XL280 gRNA, paired with 0193 (U6 promoter R) |
| ZhaoLab0275/YZ | AGAGTGCGAGGGTTAGTAGG | *RTF1* deletion in XL280 Test F |
| ZhaoLab0276/YZ | GACAAGCAAAGCCCGAGT | *RTF1* deletion in XL280 Test R |
| ZhaoLab0306/YZ | GTGGCGGTGGGCCGGCCTCTGACCTCGAGAACGAGCTTT | *RTF1* forward with FseI for FLAG tag |
| ZhaoLab0307/YZ | ctgctactgtaacccttaatTCAGAAATCTCCCAAATCTAGATCCAGCTG | *RTF1* reverse with PacI for FLAG tag |
| ZhaoLab0177/YZ | cacagaaaacttcaaacccatggataaagcggaattaattcccgagcctccaaaaaagaagagaaaggtcTCTGAAGATGATGAACCAGC | *HMD* forward with FseI for FLAG tag |
| ZhaoLab0178/YZ | tgcgatcgcggccggccCGCTCACACTAGTGTGCTTG | *HMD* reverse with SmaI for FLAG tag |
| ZhaoLab0310/YZ | cacagaaaacttcaaacccatggataaagcggaattaattcccgagcctccaaaaaagaagagaaaggtcGCGCCCAACAGGGCCGA | *Plus3* reverse with FseI for FLAG tag |
| ZhaoLab0311/YZ | tgcgatcgcggccggcccAGCCTTTATTTCATCGTGCT | *Plus3* reverse with Sma for FLAG tag |
| ZhaoLab1054/YJ | agaaaacttcaaaggccggcccATGTCTGACCTCGAGAACG | *RTF1* forward with FseI for mNeonGreen tag |
| ZhaoLab1055/YJ | cccttggacaccattgcgatGAAATCTCCCAAATCTAGAT | *RTF1* reverse with AsisI for mNeonGreen tag |
| ZhaoLab0267/YZ | aaacttcaaaggccggatggataaagcggaattaattcccgagcctcc | *HMD* forward with FseI for mNeonGreen tag |
| ZhaoLab0268/YZ | tggacaccattgcgatcgcGCTCACACTAGTGTGCT | *HMD* reverse with AsisI for mNeonGreen tag |
| ZhaoLab0315/YZ | ctcggtacccggggcggccgcgagctcggcagatacgatatgttg | RGS2membrane Forward with NotI for mNeonGreen tag |
| ZhaoLab0316/YJ | tggacaccatGGGCCCagaaccacttccgcccga | RGS2membrane reverse with ApaI for mNeonGreen tag |
| ZhaoLab1047/YJ | CGGTGGCTCTGGGCCGGCCaggagcatgatccgatacat | *RTF1* forward with FseI for RGS2-mNeonGreen tag |
| ZhaoLab1048/YJ | ctgctactgtaacccttaattaaGAAATCTCCCAAATCTAG | *RTF1* reverse with AsisI for RGS2-mNeonGreen tag |
| ZhaoLab1049/YJ | gctactgtaacccttaattaaGCTCACACTAGTGTGCTTG | *HMD* reverse with AsisI for mNeonGreen tag |
| ZhaoLab0872/YJ | GGAAATCGAGAGAGCAAACATCTTGGCGT | Primer for RTF1^E95A^ (GAA-GCA) |
| ZhaoLab0873/YJ | ACGCCAAGATGTTTGCTCTCTCGATTTCC | Primer for RTF1^E95A^ (GAA-GCA) |
| ZhaoLab0874/YJ | AGCGCTTGATGCGATGGCCAAGACTGCTCATGGT | Primer for RTF1^F118A^ (UUC-GCC) |
| ZhaoLab0875/YJ | ACCATGAGCAGTCTTGGCCATCGCATCAAGCGCT | Primer for RTF1^F118A^ (UUC-GCC) |
| ZhaoLab0823/SW | TTGGAAAGTGCGAGGGTT | *RTF1* deletion in H99 Test F |
| ZhaoLab0824/SW | GATTATGTCGGAGTTGAGC | *RTF1* deletion in H99 Test R |
| ZhaoLab0792/RL | CTCTGGTTGGCACGGTG | *real time primer for testing JEC21 DNase effect* |
| ZhaoLab0793/RL | CGTCGGTCAATCTTCTCG | *real time primer for testing JEC21 DNase effect* |
| ZhaoLab0794/RL | CGTCACCACTGAAGTCAAGT | *TEF1 real time primer* |
| ZhaoLab0795/RL | AGAAGCAGCCTCCATAGG | *TEF1 real time primer* |
| ZhaoLab0593/YJ | AATGGTGGCACGAACGATCT | *CFL1 real time primer* |
| ZhaoLab0594/YJ | GTTGTCGCAATCGGGTTCAG | *CFL1 real time primer* |
| ZhaoLab0595/YJ | GTGATGACGACAAGGAGGCTGTT | *FAD1 real time primer* |
| ZhaoLab0596/YJ | GAGACGCCAGGGATGTTGATGAA | *FAD1 real time primer* |
| ZhaoLab0599/YJ | CAGGGTTGTAAGTTCGTTCG | *FAS1 real time primer* |
| ZhaoLab0600/YJ | TCGCGACTCCTCGAAAT | *FAS1 real time primer* |
| ZhaoLab0605/YJ | GCCATCTTACCCCTACCATCTAC | *ZNF2 real time primer* |
| ZhaoLab0606/YJ | TGGACATAGGAACGCTGACAAT | *ZNF2 real time primer* |
| ZhaoLab0601/YJ | TAGCGGAGCGGACTGGAAAGA | *STE3alpha real time primer* |
| ZhaoLab0602/YJ | CTCGACCGAGACGGCAATCATTA | *STE3alpha real time primer* |
| ZhaoLab0603/YJ | GCGAATCCACCACCGAATCAATC | *STE6 real time primer* |
| ZhaoLab0604/YJ | CGACGACTGCAACGCACTCT | *STE6 real time primer* |
| ZhaoLab0607/YJ | ATCTTCACCACCTTCACTTCT | *MFalpha2 real time primer* |
| ZhaoLab0608/YJ | CTAGGCGATGACACAAAGG | *MFalpha2 real time primer* |
| ZhaoLab1252/YJ | GCTCCTCGCTACATCTCCTCA | *MAT2 real time primer F* |
| ZhaoLab1253/YJ | TGTTTCGGTCTACGATACCAGTT | *MAT2 real time primer R* |
| ZhaoLab1254/YJ | TTGTTGGAGGATTTCAGGTTGA | *PUM1 real time primer F* |
| ZhaoLab1255/YJ | GTCTTCAGGAGTGGCGGTTT | *PUM1 real time primer R* |

| **Antibody** | **Item number** | **Dilution** | **Brand** |
| --- | --- | --- | --- |
| Ubiquityl-Histone H2B-K120 Rabbit mAb | 5546T | 1:1000 | Cell signaling |
| TriMethyl-Histone H3-K4 Rabbit pAb | A2357 | 1:2000 | ABclonal |
| DiMethyl-Histone H3-K4 Rabbit pAb | A2356 | 1:2000 | ABclonal |
| MonoMethyl-Histone H3-K4 Rabbit pAb | A2355 | 1:2000 | ABclonal |
| Histone H3 Rabbit pAb | A2348 | 1:5000 | ImmunoWay |
| Flag-Tag Mouse mAb | AB0008 | 1:2000 | Abways |
| Goat Anti-Mouse | RS0001 | 1:20000 | ImmunoWay |
| Goat Anti-Rabbit | RS0002 | 1:20000 | ImmunoWay |
